# Effects of ultrasonic pretreatment on physicochemical properties and drying behavior of *Cunninghamia lanceolata* and *Eucalyptus grandis × urophylla*

**DOI:** 10.1016/j.ultsonch.2025.107549

**Published:** 2025-09-03

**Authors:** Jing Qian, Taoyu Han, Taorong Cheng, Pengfei Xia, Zekai Sun, Chunping Dai, Jiejie Sun

**Affiliations:** a Lab of Wood Quality Improvement & High Efficient Utilization, Engineering Center for Bamboo-Based Plastic Substitution, School of Materials and Chemistry, Anhui Agricultural University, Hefei 230036 Anhui, China; b Faculty of Forestry, University of British Columbia, Vancouver, BC, Canada

**Keywords:** Ultrasonic pretreatment, Wood drying, Wood pore, Physicochemical properties, *Cunninghamia lanceolata*, *Eucalyptus grandis × urophylla*

## Abstract

Ultrasonic treatment offers promising potential for improving wood drying efficiency and permeability, yet its effects on the coordinated structural and drying responses of different species remain underexplored. This study assessed the impact of 1–5 h ultrasonic treatments (320 W, 40 kHz) on the physicochemical properties, pore structure, permeability, and drying behavior of *Cunninghamia lanceolata* and *Eucalyptus grandis × urophylla*. Results showed that ultrasonic pretreatment did not alter cellulose crystallinity type but reduced crystallinity and free hydroxyl content, while increasing mass loss, microfibril angle, and hygroscopic dimensional stability. With increasing treatment time, cell double wall thickness initially decreased and then stabilized, and Shore hardness declined across all three sections. SEM and MIP analyses revealed that ultrasound gradually disrupted pit membranes, increased the proportion of macro- and mesopores, total pore volume, and porosity, thereby enhancing gas permeability. Treatments of 1–4 h showed the most significant improvements, while excessive treatment (5 h) led to structural collapse or pore closure. Permeability was enhanced by up to about 63 % and 60 % in radial and tangential directions of *C. lanceolata*, and by about 42 % and 33 % in *E. grandis*, respectively. Drying time was shortened, and drying rate and diffusion coefficient were increased, while volumetric shrinkage was reduced. Drying rates were raised by about 10 % and 13 % after 2 h (*C. lanceolata*) and 4 h (*E. grandis*) treatments, respectively. However, bound water was found to be less responsive to ultrasonic stimulation than free water. These findings provide a theoretical and practical basis for optimizing ultrasonic parameters to improve the drying performance and permeability modification of fast-growing plantation wood.

## 1 Introduction

Wood drying is a critical stage in wood processing, directly influencing the dimensional stability, mechanical performance, and overall economic value of the final product [1]. The efficiency and quality of drying largely depend on the wood’s permeability, which determines both the rate and uniformity of moisture migration. This permeability is closely linked to species-specific anatomical characteristics, including the presence and distribution of vessels, tracheids, and pits, as well as variations in heartwood/sapwood ratios, chemical composition (e.g., cellulose, hemicellulose, lignin, and extractives), and physical properties like grain orientation and density [2]. Although wood is inherently a porous material, high porosity does not necessarily equate to good permeability, as effective fluid transport relies on the connectivity of microscopic structures, particularly the integrity of pit membranes and the size and distribution of ultra-micropores [3,4]. To understand the mechanisms underlying moisture migration, it is essential to distinguish between pathways above and below the fiber saturation point (FSP). Above the fiber saturation point (FSP), water exists as free water and primarily migrates through the capillary network formed by cell lumina, vessels, and tracheids. This movement is constrained by the connectivity of pit structures, the aspiration of pit membranes, and the deposition of extractives [5,6]. Below the FSP, water is present as bound water within the micropores of the cell wall, and its movement is governed by diffusion mechanisms through these ultra-micropores, which are influenced by factors such as cell wall thickness and pore size distribution [7,8]. Therefore, the continuity of the pit system and the microstructural characteristics of the cell wall are key anatomical determinants regulating moisture transport efficiency during the wood drying process.

Given the inherent limitations in wood permeability, various techniques have been explored to enhance moisture transport and improve drying performance, including biological treatments (e.g., bacteria, enzymes, fungi), chemical pretreatments (e.g., acid-base treatment, swelling agents), physical modifications (e.g., high-temperature steam, microwave irradiation, freeze–thaw cycles) [[9], [10], [11], [12], [13], [14]]. Among these, physical methods have attracted growing attention due to their environmentally friendly nature and controllability. In recent years, ultrasonic treatment has emerged as a promising non-thermal intensification technique, owing to its combined effects of acoustic cavitation, microjets, and mechanical vibration [15]. It has demonstrated success in diverse fields such as food dehydration, biomass processing, and active compound extraction [[16], [17], [18]]. In the context of wood processing, studies have shown that ultrasound can disrupt sealed pit membranes and extractive blockages at the microscale, enhance connectivity between cell lumina, rearrange the orientation of cell wall components, and even weaken the hydrogen bonding between cellulose, hemicellulose, and lignin [[19], [20], [21]]. These mechanisms collectively improve cell wall permeability and accelerate moisture transport. For instance, He et al. [22] applied ultrasound at 10 W/cm^2^ and frequencies of 28 and 40 kHz for 30–90 min to saturated *Populus cathayana* specimens, followed by vacuum drying at 40 °C and 0.08 MPa. The water diffusion coefficient increased by 22 % at 28 kHz for 30 min and by 50 % at 40 kHz for 80 min. Liu et al. [23] treated *Eucalyptus urophylla × E. grandis* with ultrasound at 400 W for 1, 3, and 6 h, which improved drying rates by up to 28 % below 24 % MC and reduced total shrinkage by 14.9 % after a 3 h treatment. These improvements were attributed to structural changes such as pit membrane rupture and microstructural rearrangement.

However, most existing studies have focused on single species, or localized structural changes. A comprehensive understanding of the coordinated structure-performance responses across different wood types and treatment times remains lacking. Therefore, *Cunninghamia lanceolata* (a representative softwood) and *Eucalyptus grandis × urophylla* (a representative hardwood) were selected in this study to systematically examine the effects of ultrasonic treatment durations (1–5 h) on physicochemical properties, pore structure, gas permeability, and drying behavior. The objective was to elucidate species-specific mechanisms of structural regulation and drying response under ultrasonic stimulation. This research is expected to provide a theoretical foundation for developing optimized ultrasound parameters tailored to wood anatomical characteristics, and to offer technical support for upgrading drying processes and improving the utilization efficiency of fast-growing plantation wood.

## 2 Materials and methods

### 2.1 Materials

In this study, specimens of *Cunninghamia lanceolata* and *Eucalyptus grandis × urophylla* were selected as the experimental materials. The samples were provided by the Guangxi Academy of Forestry, and the trees were approximately 15 years old. The initial moisture content of *C. lanceolata* was approximately 11.81 %, with an air-dry density of 0.308 g/cm^3^, whereas *E. grandis × urophylla* exhibited a higher initial moisture content of approximately 18.98 % and an air-dry density of 0.682 g/cm^3^. All specimens were processed into uniform dimensions of 50 mm × 50 mm × 20 mm (longitudinal × tangential × radial). The primary chemical reagents used in this study included deionized water (purified using a Milli-Q system), absolute ethanol (analytical grade, AR ≥ 99.7 %, Aladdin Biochemical Technology Co., Ltd., Shanghai, China), anhydrous ethylenediamine (AR ≥ 99.5 %, Suyi Chemical Reagent Co., Ltd., Shanghai, China), polyethylene glycol (Mn = 4000, Aladdin Biochemical Technology Co., Ltd.), impact-resistant polystyrene (Aladdin Biochemical Technology Co., Ltd.), and butyl acetate (AR ≥ 99 %, Aladdin Biochemical Technology Co., Ltd.). All chemicals were of analytical grade and used without further purification.

### 2.2 Preparation of samples

For each species, a total of 110 specimens were prepared and randomly divided into 11 groups, with 10 specimens per group. All specimens were immersed in deionized water for 10 days to adjust the moisture content to about 75 %. The control group was left untreated, while the remaining 100 specimens were subjected to ultrasonic treatment using an ultrasonic cleaner (Suzhou Jiangdong Precision Instrument Co., Ltd.) at a frequency of 40 kHz and power of 320 W, with treatment durations of 30, 60, 90, 120, 150, 180, 210, 240, 270, and 300 min, respectively. During treatment, specimens were fixed at a position 2 cm above the ultrasonic transducer.

### 2.3 Determination of mass loss rate

Five specimens were selected from each group and oven-dried at 103 ± 2 °C until constant weight, requiring approximately 50 h for *C. lanceolata* and 78 h for *E. grandis × urophylla*. The oven-dry mass before ultrasonic treatment (*M_0_*) was estimated based on the initial air-dry mass and corresponding moisture content of each specimen. After ultrasonic treatment, the specimens were again oven-dried, and their final oven-dry mass (*M_1_*) was recorded. The mass loss rate (*ML*, %) was then calculated as Equation (1), and the final result for each group was expressed as the average of the five replicates.

(1) $$\text{WL=}\frac{{\text{W}}_{\text{0}}\text{-}{\text{W}}_{\text{1}}}{{\text{W}}_{\text{0}}}\times 100\%$$

### 2.4 Attenuated total reflectance − Fourier transform infrared spectroscopy (ATR-FTIR)

After oven-drying, specimens from both the ultrasonic treatment and control groups were ground into powder using a high-speed grinder and passed through a 100–120 mesh sieve. The appropriate powder of each wood (approximately 2 mg) was used for Fourier transform infrared (FTIR) spectroscopy. The measurements were performed using a Tensor II spectrometer (Bruker Optics, Ettlingen, Germany) equipped with a diamond attenuated total reflectance (ATR) accessory (iD7, Thermo Scientific). Before each measurement, the ATR crystal surface was thoroughly cleaned with ethanol to eliminate potential cross-contamination. A background spectrum was collected prior to each sample scan. Spectra were acquired over the mid-infrared range from 4000 to 500 cm^−1^, at a resolution of 0.482 cm^−1^. For each sample, 32 scans were accumulated to improve the signal-to-noise ratio. All measurements were conducted in triplicate, and the spectra were averaged for subsequent analysis to assess changes in chemical functional groups resulting from ultrasonic treatment.

### 2.5 Determination of microfibril angle (MFA)

Specimens were cut into thin slices measuring 20 mm × 10 mm × 1 mm (L × T × R) using a rotary microtome (KD1508A, Jinhua Kedi Instrument Co., Ltd., Jinhua, China). The surface of each wood slice was polished using P1200 grit sandpaper until smooth and flat. The prepared specimens were then mounted on the rotating sample stage of the XD-6 X-ray diffractometer for measurement. The test conditions were set as follows: Cu Kβ radiation (λ = 1.54056 Å), 36 kV voltage, 20 mA current, a rotation angle range of 90° to 360°, and a scanning speed of 16°/min. Each group was tested in triplicate to ensure repeatability and accuracy. The diffraction data were processed and analyzed using Origin software, and the microfibril angle (MFA) of samples was calculated using the 0.6  T method, based on 60 % of the full width at half maximum (FWHM) of the (002) diffraction peak.

### 2.6 Cell wall thickness measurement

Wood specimens were softened using ethylenediamine (EDA) treatment followed by polyethylene glycol (PEG) embedding to ensure structural integrity during sectioning. Transverse sections with a thickness of 14  μm were prepared using a rotary microtome (Leica RM2016, Leica Biosystems, Nussloch, Germany). The sections were mounted on glass slides without staining and imaged under an upright fluorescence microscope (Y-TV55, Nikon Corporation, Tokyo, Japan). Images were captured at appropriate magnifications to clearly visualize the cell wall boundaries. Cell wall thickness was measured using ImageJ software (National Institutes of Health, USA), based on calibrated scale bars. For each group, 50 individual measurements were conducted to ensure statistical reliability.

### 2.7 Shore D hardness

The Shore D hardness of oven-dried wood specimens, both untreated and ultrasonically treated, was measured using a Shore D durometer (Wenzhou Haibao Instrument Co., Ltd., China) equipped with a conical indenter tip of 0.2  mm diameter. Measurements were conducted on all three anatomical sections (cross, radial, and tangential) of the specimens to evaluate anisotropic hardness behavior. For each treatment group, 10 replicate specimens were tested. On each sectional surface, three measurement points were randomly selected and tested to account for within-sample variability. The Shore hardness (*HD*) value of each specimen was determined as the average of these three readings. The Shore D hardness was calculated according to the following formula:

(2) $$\mathrm{HD}=\frac{100-L}{0.025}\times 100\%$$

where *L* is the displacement (mm) of the indenter relative to the presser foot.

### 2.8 Hygroscopic dimensional stability

The test was conducted following Chinese National Standard LY/T 2490–2015 (Test Method for Dimensional Stability of Modified Wood). The initial oven-dried mass, along with radial, tangential, and longitudinal dimensions, were measured for each specimen. The samples were then placed in a controlled environment at 25 °C and approximately 75 % relative humidity (simulated using a saturated NaCl solution) until they reached moisture equilibrium. After equilibration, the final mass and dimensional values in all three directions were recorded. In each group, ten randomly selected specimens were measured in triplicate. The mean of the three measurements was used as the value for each specimen, and the group result was calculated as the average of these ten values. The moisture content (*MC*, %), and tangential/radial swelling rate (*ST*, %) were calculated using the following equations:

(3) $$\text{MC=}\frac{{\text{W}}_{\text{6}}\text{-}{\text{W}}_{\text{5}}}{{\text{W}}_{\text{5}}}\times 100\%$$

where *W_6_* is the mass after moisture equilibration (g), and *W_5_* is the oven-dried mass (g).

(4) $$\text{ST}=\frac{L_{2}-L_{1}}{L_{1}}\times 100\%$$

where *L_2_* is the tangential/radial dimension after moisture equilibration (mm), and *L_1_* is the oven-dried tangential/radial dimension (mm).

### 2.9 X-ray diffraction analysis (XRD)

Wood powder samples (100–120 mesh, oven-dried) from each treatment group were pressed into thin disks at room temperature for X-ray diffraction (XRD) analysis. The measurements were conducted using a multifunctional powder X-ray diffractometer (Model XD-6, Purkinje General Instrument Co., Ltd., Beijing, China) equipped with a Cu target X-ray tube and a curved graphite crystal monochromator. The instrument was operated under the following conditions: tube voltage of 40 kV and tube current of 20 mA; divergence slit (DS) and scatter slit (SS) were both set to 1°, while the receiving slit (RS) and receiving slit module (RSM) were set to 0.15 mm and 0.45 mm, respectively. A scintillation counter was used as the detector. The scan was performed over a 2θ range of 5° to 40° in continuous mode, with a step size of 0.04° and a scanning speed of 2°/min. The diffraction intensity was recorded in real-time and plotted as a function of the 2θ angle. Each group was tested in triplicate to ensure repeatability and accuracy. The resulting XRD patterns were analyzed using Origin software, and the relative crystallinity index (*CrI*, %) was calculated using the Segal method [24]:

(5) $$\text{CrI=}\frac{I_{002}-I_{\mathrm{am}}}{I_{002}}\times 100\%$$

where *I_002_* is the intensity of the 002 lattice diffraction peak**,** representing the crystalline region of cellulose, and *I_am_* is the intensity of the amorphous background, typically taken at the minimum intensity near 2θ = 18°.

### 2.10 Scanning electron microscopy (SEM) observation

To examine the microstructural changes in cell lumens and pit structures after ultrasonic treatment, SEM observations were performed on the radial section of both untreated and ultrasonically treated wood specimens. Samples were prepared using a rotary microtome (RM2265, Leica Microsystems, Wetzlar, Germany) by slicing each specimen (freeze-dried) into tangential and radial sections with dimensions of approximately 10 mm × 10 mm × 1 mm (L × R × T). Prior to imaging, all samples were sputter-coated with a thin layer of gold for 60 s using an ion sputter coater (SBC-12, Beijing Zhongke Keyi Technology Co., Ltd., Beijing, China) to enhance surface conductivity and prevent charging during observation. SEM imaging was performed using a tungsten filament scanning electron microscope (VEGA3, TESCAN, Brno, Czech Republic) operating at an accelerating voltage of 10 kV.

### 2.11 Gas permeability test

Specimens measured 10 mm × 20 mm × 2 mm (L × T × R) for the tangential direction and 10 mm × 2 mm × 20 mm (L × T × R) for the radial direction, respectively. For each group, 10 replicate specimens were prepared to ensure statistical reliability. After ultrasonic treatment, all specimens were conditioned at 20 ± 2 °C and 65 ± 5 % relative humidity until they reached a moisture content of 9 % ± 1 %, as gas permeability is highly sensitive to variations in moisture content. This conditioning ensured consistent test conditions across all treatments. Gas permeability was measured using a custom-built gas permeability apparatus (self-make, shown as Fig. S1). Each specimen was securely mounted in the holder to prevent leakage during testing. The vacuum pump was activated to draw gas through the sample. Once the pressure differential and gas flow rate readings stabilized, the data were recorded. The procedure was repeated for both tangential and radial orientations to evaluate anisotropic permeability behavior. The gas permeability coefficient was calculated using the following equation (4):

(6) $${\text{K}}_{\text{G}}=\frac{\text{QLP}\eta }{\text{A}\Delta \text{P}{\text{P}}_{\text{average}}}$$

where *K_G_* represents the gas permeability coefficient (m^2^), η is the dynamic viscosity of air, taken as 1.8E-05 Pa·s, *Q* is the volumetric gas flow rate (cm^3^/s), *L* is the length of the gas flow path through the specimen (cm), *A* is the cross-sectional area available for gas flow (cm^2^), *P* is the pressure measured at the gas flowmeter (Pa), and *ΔP* is the pressure difference across the specimen (Pa).

### 2.12 Mercury intrusion porosimetry (MIP) for wood pore structure analysis

Mercury intrusion porosimetry (MIP) was performed using a PoreMaster 33GT porosimeter (Quantachrome, USA) to evaluate the pore structure of wood after ultrasonic treatment. Cubic specimens (6 mm × 6 mm × 6 mm, T × R × L) were sectioned from the tangential face, ∼2 cm from the longitudinal axis near the ultrasonic horn, to ensure sampling consistency across groups. Prior to testing, the specimens were oven-dried at 103 ± 2 °C for at least 24 h to remove all moisture, as residual water could interfere with mercury intrusion results. Each dried specimen was loaded into a 0.5 cm^3^ × 1.5 in. cylindrical penetrometer. The dry mass of each specimen was recorded before mercury filling. The intrusion procedure included both low- and high-pressure stages. During testing, mercury was incrementally forced into the specimen pores under controlled pressure. The total intrusion volume, incremental volume, and cumulative intrusion curve were automatically recorded by the instrument software. Pore size distribution was calculated using the Washburn equation, which relates the applied pressure (p) to the corresponding pore radius (r) as follows:

(7) $$r=\frac{2\gamma cos\theta }{p}$$

where *r* is the pore radius (m), *γ* is the surface tension of mercury (0.48 N/m), *θ* is the contact angle of mercury (141°), and *p* is the applied pressure (Pa).

### 2.13 Drying performance

To simulate practical drying conditions, both end faces of the specimens were sealed with heat-resistant, impermeable aluminum foil tape and wrapped with plastic film to minimize early-stage evaporation. The specimens were then placed in a 60 °C constant-temperature drying oven. Mass was recorded at 1-hour intervals until the moisture content dropped to approximately 10 %. Afterwards, the specimens were transferred to a 103 ± 2 °C oven and dried to a constant mass. The final oven-dry mass and three-dimensional dimensions at the marked positions were recorded for further analysis. *MC* at time t (h) was calculated by:

(8) $${\text{MC}}_{\text{t}}=\frac{{\text{W}}_{\text{t}}\text{-}{\text{W}}_{\text{0}}}{{\text{W}}_{\text{0}}}\times 100\%$$

where *W_t_* is the sample weight at time t, and *W_0_* is oven-dry weight.

Drying rate (*DR*, %/h) was calculated as:

(9) $$\text{DR=}\frac{{\text{MC}}_{\text{initial}}\text{-}{\text{MC}}_{\text{final}}}{\text{t}}$$

where *MC_initial_* and *MC_initial_* (%) is the MC before dying and MC at time t (h), respectively.

The MC at fiber saturation point (*M_FSP_*) was determined using following equation:

(10) $${\text{M}}_{\text{FSP}}\text{=0.3-0.001(T-20)}$$

where *T* (60 °C) is the temperature. And at 60 °C, *M_FSP_* was about 26 %.

Moisture diffusion coefficient (D_e_) was estimated by:

(11) $$D_{e}=\frac{kL^{2}}{\pi^{2}}$$

where *k* is the drying constant (s^−1^) and *L* is specimen thickness (m).

Volume shrinkage rate (*VS*, %) was calculated by:

(12) $$\mathrm{VS}=(1-\frac{S_{1}}{S_{0}})\times 100\%$$

where *S_1_* is the dried volume and *S_0_* is the original volume, cm^3^.

## 3 Results and discussion

### 3.1 Physical, chemical, morphology and microstructural responses

**Fig. 1:**
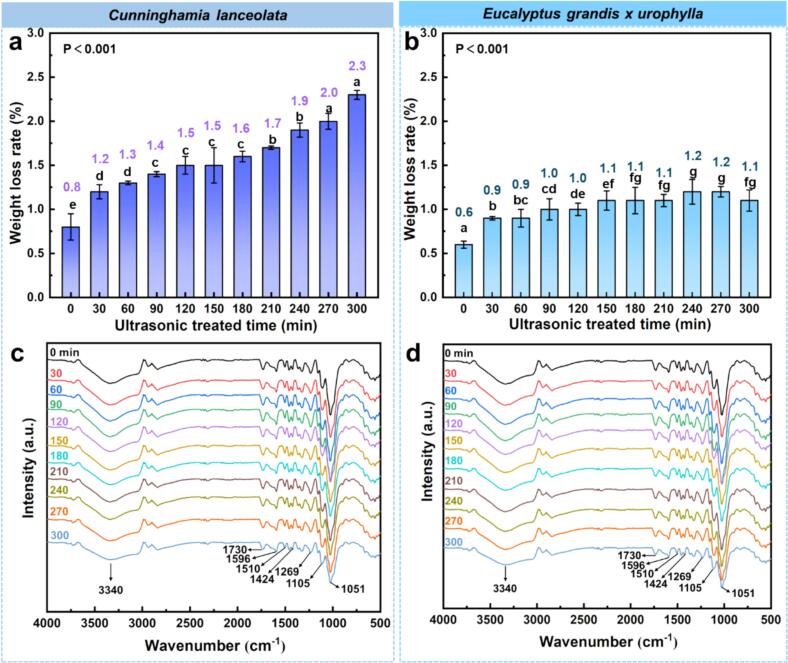
Mass loss and FTIR spectra of *C. lanceolata* and *E. grandis × urophylla* after ultrasonic treatment. (a) Mass loss rate of *C. lanceolata*, (b) Mass loss rate of *E. grandis × urophylla,* (c) FTIR spectra of *C. lanceolata*, (d) FTIR spectra of *E. grandis × urophylla*.

As shown in Fig. 1, ultrasonic pretreatment significantly increased the mass loss rates of *C. lanceolata* (Fig. 1a) and *E. grandis × urophylla* (Fig. 1b), with notable differences among treatment durations. For *C. lanceolata*, the control group had a mass loss of approximately 0.8 %, primarily caused by water-soluble extractives removed during prior soaking. With increasing ultrasonic duration, the mass loss rose steadily, reaching 2.3 % at 300 min, about 1.50 % higher than the untreated group. A similar trend was observed in *E. grandis × urophylla*, with control samples showing a mass loss of 0.6 %. The loss rate increased significantly up to 180 min, then plateaued between 180 and 300 min, stabilizing around 1.2 %. The highest value (1.2 %) appeared at 270 min, indicating that most water-soluble compounds were removed within the first 3 h of treatment.

Fig. 1c and 1d illustrate changes in ATR-FTIR spectra of *C. lanceolata* and *E. grandis × urophylla* under different ultrasonic durations. While the major chemical groups remained consistent, variations in peak intensity reflected structural responses in lignin, hemicellulose, and cellulose. In *C. lanceolata*, the O–H stretching band at 3340 cm^−1^ decreased initially and slightly recovered later but remained below control levels, indicating hydroxyl group reduction. The C–H bands at 2936 and 2842 cm^−1^ showed mild fluctuations, suggesting redistribution of hydrophobic groups. In the fingerprint region (1600–800 cm^−1^), lignin-related peaks at 1596, 1510, and 1269 cm^−1^ intensified with treatment, indicating increased exposure or partial depolymerization of lignin. The 1105 cm^−1^ peak (G units) first rose then declined, while the hemicellulose-related 1730 cm^−1^ peak increased after 120 min. Cellulose-associated peaks at 1051 and 896 cm^−1^ showed slight decreases or stabilization, suggesting minimal disruption to its backbone. *E. grandis × urophylla* exhibited similar overall trends, though with species-specific responses. The 3340 cm^-1^ O–H peak also showed a “decrease-then-increase” trend. However, both C–H peaks (2936 and 2842 cm^−1^) declined, indicating a reduction in hydrophobic groups. Lignin-related peaks peaked around 120–150 min, then declined, and eventually dropped below control levels after extended treatment, suggesting partial lignin degradation. G-type lignin signals at 1109 cm^−1^ decreased steadily. Meanwhile, the 1730 cm^−1^ peak (C=O in hemicellulose) and 1157 cm^−1^ (C–O–C in cellulose) also declined after 180 min. The 896 cm^−1^ band, related to β-glycosidic linkages, weakened after 120 min, indicating ultrasonic impact on cellulose microfibril arrangement without damaging its framework.

**Fig. 2:**
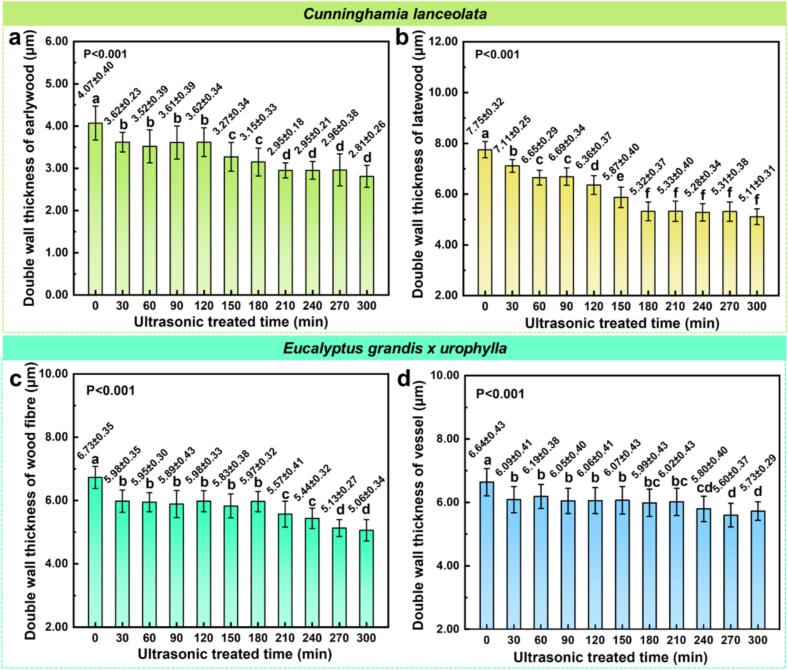
Cell wall thickness changes under ultrasonic treatment (Fig. 2a-d). a) double wall thickness of earlywood fibers in *C. lanceolata*, b) double wall thickness of latewood fibers in *C. lanceolata*, c) double wall thickness of fibers in *E. grandis × urophylla*, d) double wall thickness of vessel in *E. grandis × urophylla.*

Fig. 2 illustrates the effects of ultrasonic treatment duration on cell wall thickness in *C. lanceolata* and *E. grandis × urophylla*. One-way ANOVA revealed that ultrasonic treatment significantly affected the double wall thickness of both earlywood and latewood fibers in *C. lanceolata* (earlywood: F(10, 263) = 72.39, p < 0.001, r = 0.7, latewood: F(10, 223) = 256.90, p < 0.001, r = 0.9). In untreated samples, earlywood fibers had an average thickness of 4.07 μm, while latewood fibers were thicker at 7.75 μm. Both decreased with increasing treatment duration, showing a “decline-stabilization-further decline” pattern, reaching minimum values of 2.82 μm (−30.71 %) and 5.11 μm (−34.06 %) at 300 min, respectively. Post hoc comparisons indicated that earlywood thickness decreased significantly between 0–30 min, stabilized during 30–120 min, declined again at 150–180 min, and remained stable until a final drop at 300 min. Latewood thickness showed continuous decline from 0-180 min and plateaued thereafter. Similar trends were observed in *E. grandis × urophylla* (Fig. 2c, d). Ultrasonic treatment significantly affected the double wall thickness of fibers (F(10, 264) = 86.77, p < 0.001, r = 0.7) and vessel walls (F(10, 259) = 20.83, p < 0.001, r = 0.5). In control samples, fiber and vessel wall thickness were 6.73 μm and 6.64 μm, respectively. After 30 min of treatment, fiber thickness dropped rapidly to 5.98 μm and remained stable between 30–180 min. It further declined to 5.57–5.44 μm at 210–240 min and reached the lowest range of 5.13–5.06 μm at 270–300 min, reflecting a 24.81 % reduction. Vessel wall thickness decreased to 6.09 μm at 30 min, fluctuated slightly from 60-180 min, and dropped further to a minimum of 5.60 μm at 270 min (−15.66 %). These findings suggest that ultrasonic pretreatment disrupts the cell wall matrix, particularly in regions with thinner or more hydrated walls such as earlywood and fiber cells. The mechanical cavitation and microstreaming generated by ultrasonic waves likely promote delamination of the middle lamella or loosening of lignocellulosic bonding, thereby reducing wall rigidity and thickness over time [25,26].

**Fig.3:**
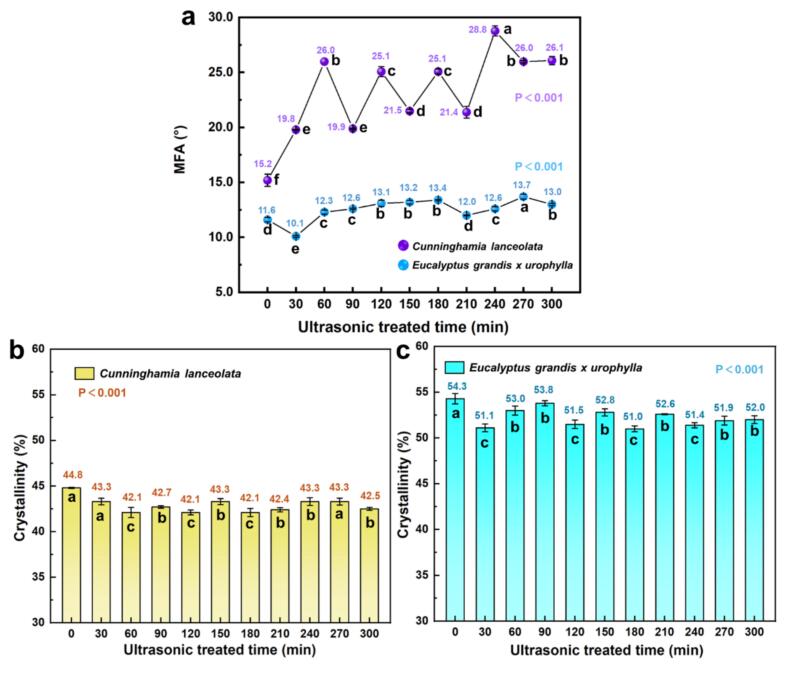
Effects of ultrasonic pretreatment duration on microfibril angle (MFA) and crystallinity of wood fibers in *C. lanceolata* and *E. grandis × urophylla*. (a) MFA variation in *C. lanceolata* and *E. grandis × urophylla*, (b) crystallinity of *C. lanceolata*, (c) crystallinity of *E. grandis × urophylla*.

Fig. 3a illustrates the effects of different ultrasonic pretreatment durations on the microfibril angle (MFA) of wood fibers in *C. lanceolata* and *E. grandis × urophylla*. For *C. lanceolata*, the MFA of untreated samples was 15.2°. Upon ultrasonic treatment, MFA increased significantly at all time points, showing an overall upward trend with fluctuations. It rose to 19.8° at 30 min, peaked at 26.0° at 60 min, and reached a maximum of 28.8° at 240 min, an increase of approximately 89.2 % over the initial value. Pearson correlation analysis revealed a significant positive correlation between treatment time and MFA (r = 0.696, p < 0.001), indicating a general increase in MFA over time. In contrast, *E. grandis × urophylla* fibers had an initial MFA of 11.6°, which decreased to 10.1° after 30 min of treatment (a 12.5 % reduction). From 60 min onward, MFA steadily increased, peaking at 13.7° at 270 min (an 18.1 % increase over the control). The pattern featured stage-wise fluctuations: increases during 60–180 min and 210–270 min, and slight declines during 0–30 min, 180–210 min, and 270–300 min, with amplitudes of 1.44°, 1.37°, and 0.61°, respectively. Overall, fluctuation amplitude decreased with time, while peak values gradually increased. Pearson correlation analysis also indicated a significant positive correlation between MFA and treatment duration (r = 0.657, p < 0.001). The increase in MFA suggests that ultrasonic treatment disturbs the microfibrillar orientation by disrupting the bonding matrix within the cell wall.

Fig. 3b and 3c show the relative crystallinity of the two species at different ultrasonic durations. As shown in Fig. 2 of the supplementary materials, all samples exhibited distinct diffraction peaks at 2θ ≈ 16° and 22°, characteristic of the cellulose I structure, indicating that ultrasonic treatment did not alter the crystalline form of cellulose. In *C. lanceolata*, the crystallinity index decreased from 44.8 % (control) to 42.1 % at 120 min (−2.7 %), then rebounded slightly and stabilized around 43.3 %, ending at 42.5 % at 300 min. In *E. grandis × urophylla*, crystallinity was initially 54.3 %, dropped to 51.0 % at 180 min (−3.3 %), and remained relatively steady between 51.4 % and 52.0 % thereafter.

Cavitation and microstreaming effects generated by ultrasonic treatment are likely to induce fibril slippage and realignment, thereby disrupting intermolecular hydrogen bonds at the crystalline-amorphous interface and leading to a reduction in crystalline integrity. Similar observations were reported by Baruah et al. [28] and Flores et al. [27], who demonstrated that ultrasonic cavitation can weaken or break hydrogen bonding between cellulose fibrils, particularly at the boundary between crystalline and amorphous regions.

### 3.2 Hygroscopic dimensional stability and Shore d hardness

**Fig. 4:**
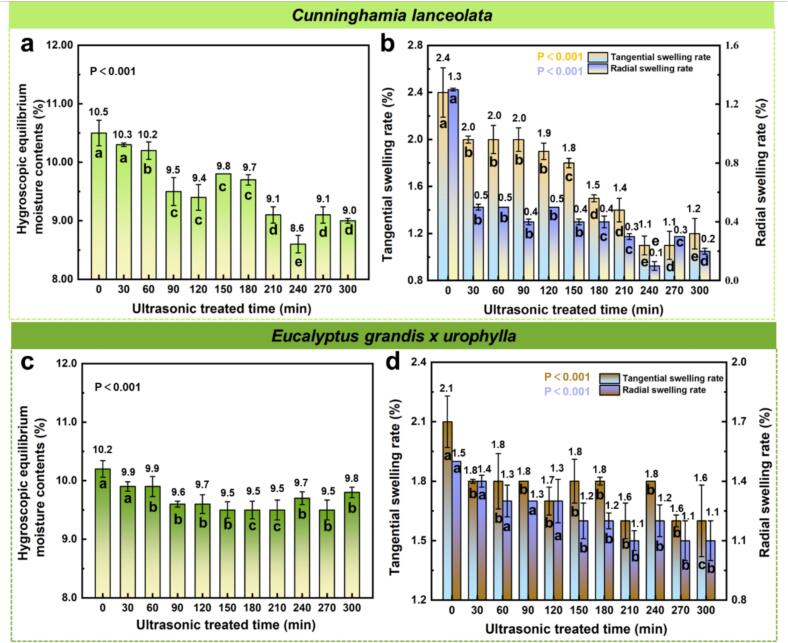
Effects of ultrasonic pretreatment time on the hygroscopic behavior and dimensional stability of *C. lanceolata* and *E. grandis × urophylla* wood under ∼ 75 % relative humidity. (a) Equilibrium moisture content of *C. lanceolata*, (b) Tangential swelling rate of *C. lanceolata*, (c) Equilibrium moisture content of *E. grandis × urophylla*, (d) Tangential swelling rate of *E. grandis × urophylla*.

Fig. 4 illustrates the effects of ultrasonic pretreatment on the equilibrium moisture content (EMC) and tangential/radial swelling ratios of *C. lanceolata* and *E. grandis × urophylla* under ∼ 75 % relative humidity. For *C. lanceolata*, the EMC of untreated samples was approximately 10.5 %. Ultrasonic treatment significantly reduced hygroscopicity, with EMC gradually decreasing over time and reaching a minimum of 8.6 % at 240 min, a 1.9 % reduction. Although minor fluctuations were observed, the overall trend remained consistently downward, indicating a reduced responsiveness to ambient humidity after treatment. In contrast, *E. grandis × urophylla* exhibited a non-linear response. Its initial EMC was 10.2 %, which declined to a minimum of 9.5 % at 210 min (−0.7 %) and then slightly increased, reaching a stable level. This pattern may reflect an initial degradation of hemicellulose that reduced hydrophilicity, followed by partial structural rearrangement of lignin or cell wall realignment, causing a slight rebound in moisture uptake. Dimensional stability also improved with ultrasonic treatment. For *C. lanceolata*, tangential swelling (Fig. 4b) decreased from 2.4 % (0 min) to 1.1 % at 240 min, while radial swelling dropped from 1.3 % to 0.1 %, showing reductions of 1.3 % and 1.2 %, respectively. *E. grandis × urophylla* (Fig. 4d) followed a similar but less pronounced trend. Tangential swelling decreased from 2.1 % to 1.6 %, with the lowest point observed at 210 min. Radial swelling dropped steadily from 1.5 % to 1.1 % over the treatment period. Ultrasonic pretreatment enhances the hygroscopic and dimensional stability of wood. This improvement can be attributed to a combination of mechanisms, including cavitation-induced loosening of the cell wall matrix, and partial degradation of hemicellulose, which reduces the content of hydrophilic groups.

Ultrasonic pretreatment enhances both hygroscopic and dimensional stability of wood, which could contribute to cavitation-induced loosening of the cell wall matrix, partial degradation of hemicellulose, lowering the abundance of hydrophilic groups and lignin reorganization, increasing cell wall rigidity and limiting dimensional changes under humidity fluctuations [29,30]. Compared to *C. lanceolata*, *E. grandis × urophylla* showed less sensitivity to ultrasonic modification, likely due to differences in cell wall ultrastructure and chemical composition.

**Fig. 5:**
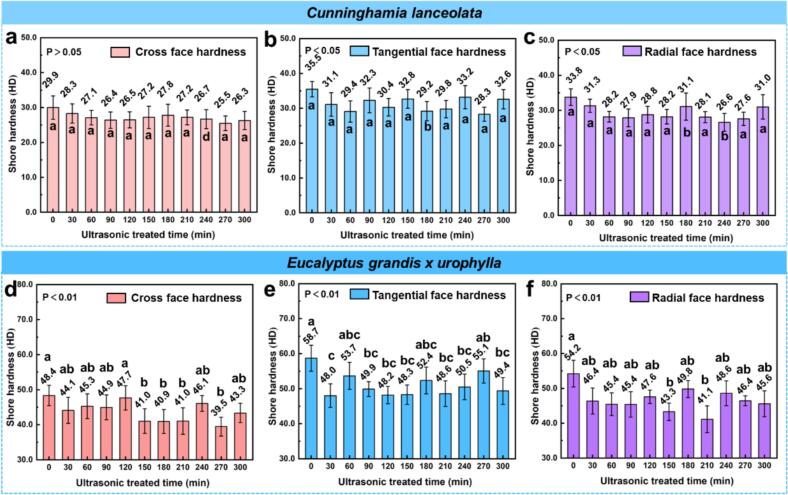
Shore D hardness of *Cunninghamia lanceolata* and *Eucalyptus grandis × urophylla* on different anatomical sections under varying ultrasonic pretreatment durations. (a–c) Cross-sectional, tangential, and radial hardness of *C. lanceolata*, (d–f) Cross-sectional, tangential, and radial hardness of *E. grandis × urophylla*.

Fig. 5 illustrates the variation in Shore D hardness on the cross, tangential, and radial surfaces of *C. lanceolata* and *E. grandis × urophylla* subjected to varying ultrasonic pretreatment durations. Across all anatomical planes, *Eucalyptus* exhibited higher initial hardness than *Cunninghamia*, consistent with its denser structure and greater lignin content [31]. One-way ANOVA revealed that ultrasonic duration had a significant effect on surface hardness in all cases (P < 0.001). For *Cunninghamia*, the untreated samples showed tangential hardness of 35.5 HD, radial 33.8 HD, and cross-sectional 29.9 HD. With increasing ultrasonic duration, all surfaces exhibited a fluctuating decreasing trend, reaching minimum values of 28.3 HD (tangential), 26.6 HD (radial), and 25.5 HD (cross-sectional) between 240–270 min. Among them, radial hardness showed the greatest decline (−21.3 %), indicating higher sensitivity to ultrasonic disruption. *Eucalyptus* followed a similar pattern. Initial values were 58.7 HD (tangential), 54.2 HD (radial), and 48.4 HD (cross-sectional), with minimum values of 48.0 HD, 41.1 HD, and 39.5 HD respectively appearing between 30–270 min. The largest reduction occurred in the radial direction (−24.1 %), again suggesting greater vulnerability in that orientation.

These reductions in Shore hardness are likely attributed to the cavitation and microstreaming effects induced by ultrasonic waves, which can generate localized shear forces, leading to delamination of cell wall layers and weakening of lignin-cellulose bonding [32]. Moreover, ultrasonic treatment may promote partial hemicellulose degradation and lignin redistribution, reducing wall rigidity and mechanical integrity [21,33]. Comparatively, the greater hardness and slower decline in *Eucalyptus* suggest a more resistant wall structure, possibly due to higher lignin cross-linking and denser cell wall ultrastructure [34]. The pronounced sensitivity of radial surfaces in both species is consistent with previous findings showing that the alignment of cellulose microfibrils and the anisotropic mechanical properties of wood make the radial direction more prone to disruption under external stress [35].

### 3.3 Pore evolution and gas permeability

**Fig. 6:**
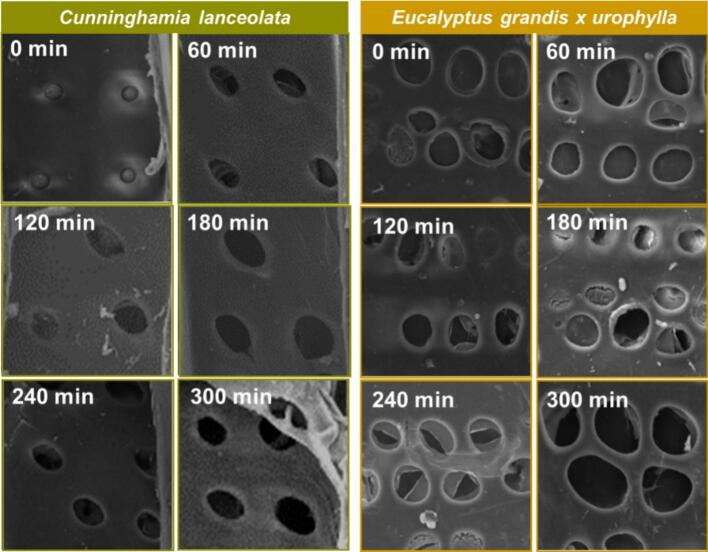
SEM micrographs under different ultrasonic treatment in *Cunninghamia lanceolata* (left) and *Eucalyptus grandis × urophylla* (right).

Fig. 6 presents scanning electron microscopy (SEM) images of *Cunninghamia lanceolata* and *Eucalyptus grandis × urophylla* subjected to various durations of ultrasonic pretreatment, focusing on changes in pit structure and vessel permeability. In untreated *C. lanceolata* samples (left column of Fig. 6), the radial sections exhibit intact, circular bordered pits with well-adhered pit membranes. No signs of detachment or rupture were observed, indicating a native, undisturbed ultrastructure. These structures are crucial for water and nutrient transport in conifers. Notably, the torus-margo structure in bordered pits allows lateral displacement of the torus under pressure differentials, enabling pit aspiration and limiting permeability, a key reference point for assessing structural alterations. Upon ultrasonic treatment, progressive damage to pit membranes was evident. During early treatment stages (30–60 min), pit membranes exhibited minor cracking and partial detachment, though overall morphology remained identifiable. With prolonged exposure (90–150 min), the membranes became extensively ruptured or fully detached. After 180–300 min of treatment, pit membranes were no longer visible, leaving open pit apertures with clearly defined borders. Similar phenomena were observed in *E. grandis × urophylla* (right column of Fig. 6). At 30 min, small cracks emerged; by 60–90 min, extensive rupture had occurred; between 120–210 min, the cracks traversed the full membrane span. By 240–300 min, the pit membranes were completely detached, and the pits appeared fully open. These changes suggest that ultrasonic cavitation selectively disrupts pit membranes, significantly enhancing liquid permeability. This aligns with prior observations that ultrasonic cavitation facilitates the removal or rupture of internal pit barriers, improving fluid transport properties [36,37]. These findings collectively demonstrate that ultrasonic pretreatment can modify both the structural integrity of pits and the overall permeability of hardwood and softwood tissues, consistent with previous reports on ultrasonic-induced delamination and extractive removal in lignocellulosic materials [19,38].

**Fig. 7:**
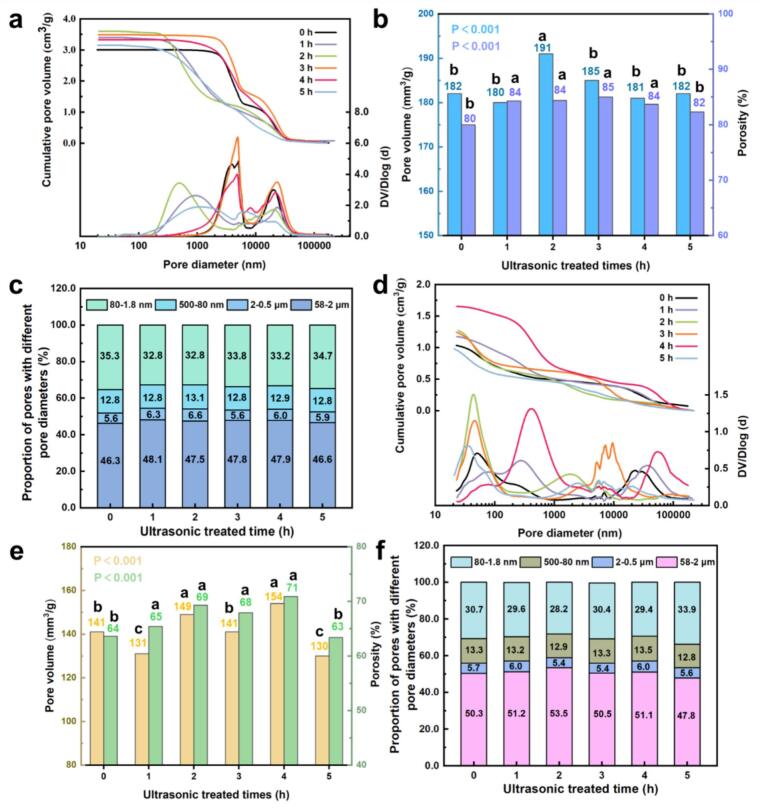
Effects of ultrasonic pretreatment duration on pore structure characteristics of *C. lanceolata* (a-c) and *E. grandis × urophylla* (d-f) as determined by mercury intrusion porosimetry (MIP). a, d) cumulative pore volume and Dv/Dlog curves. b, e) total pore volume and porosity. c, f) volume fractions of macro-, meso-, and micropores.

**Fig. 8:**
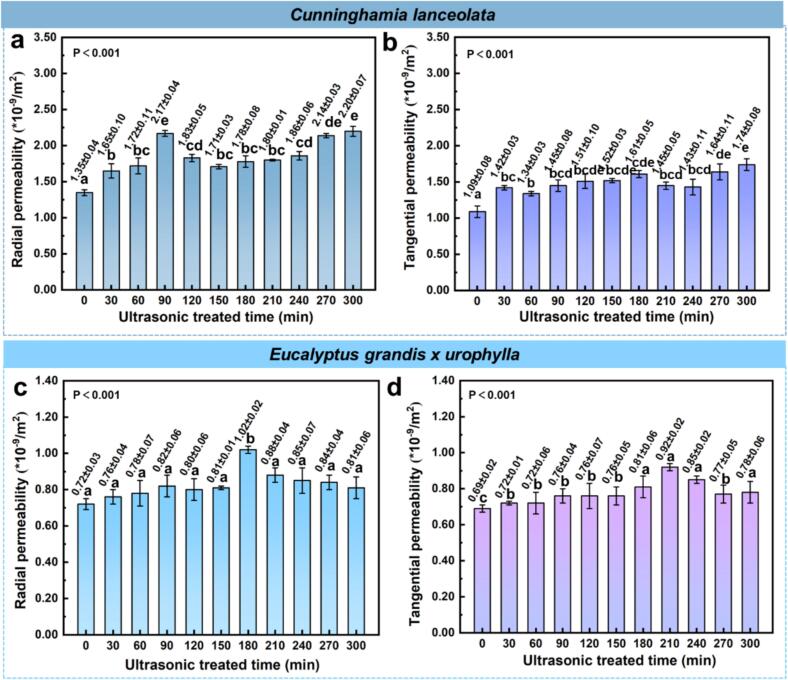
Effects of ultrasonic pretreatment duration on radial and tangential permeability of *Cunninghamia lanceolata* and *Eucalyptus grandis × urophylla*.

Fig. 7 illustrates the changes in pore size distribution of *Cunninghamia lanceolata* and *Eucalyptus grandis × urophylla* under different ultrasonic pretreatment durations. According to the pore classification system proposed by Plötze and Niemz [40], wood pores can be categorized as macropores (≥20.5 μ m), mesopores (50–80 nm), and micropores (0.8–1.8 nm). Fig. 7a and 7d show the cumulative pore volume and Dv/Dlog pore size distribution curves. In *C. lanceolata* (Fig. 7a), the untreated sample exhibited pore size peaks at 3.85, 5.07, and 20.53 μm. After ultrasonic pretreatment, the main peaks shifted toward larger pore sizes (20–23 μm), with the 3 h treatment group reaching the highest value (23.34 μm). In the small-pore region (<250 nm), cumulative pore volumes in all treated groups increased relative to the control, indicating that ultrasonic treatment facilitates microstructural loosening and enhanced pore connectivity by disrupting cell wall integrity. *In E. grandis × urophylla* (Fig. 7d), the macropore peak shifted from 22.89 μm (untreated) to the 35–52 μm range after ultrasonic treatment. Multiple new peaks emerged below 1 μm (e.g., 43.79 nm, 77.76 nm, 280.38 nm), especially in the 4 h treatment group, which exhibited the most developed pore structure. In contrast, the 5 h treatment group showed decreased peak intensity and lower cumulative pore volume across the entire pore size range, suggesting potential cell collapse or pore closure (Fig. 8)

Fig. 7b and 7e summarize changes in pore volume and porosity. In *C. lanceolata* (Fig. 7b), porosity increased from 80 % (untreated) to a maximum of 85 % after 1–4 h of treatment, before declining slightly to 82 % at 5 h. A similar upward trend was observed in pore volume. In *E. grandis × urophylla* (Fig. 7e), porosity increased from 64 % (0 h) to 71 % after 4 h of treatment, but dropped to 63 % at 5 h. Fig. 7c and 7f present the relative volume fractions of macro-, meso-, and micropores. In the untreated state, *C. lanceolata* showed 51.9 % macropores, 12.8 % mesopores, and 35.3 % micropores, while *E. grandis × urophylla* had 56.0 %, 13.3 %, and 30.7 %, respectively. Ultrasonic treatment significantly altered the pore structure of both species by increasing the proportion of macro- and mesopores and decreasing micropore content. In *C. lanceolata*, macropore content increased by 1.5–2.4 % after 1–4 h of treatment, while mesopore content showed only slight fluctuations (0.0–0.4 %) and micropores decreased accordingly. *E. grandis × urophylla* responded more strongly, with the highest macropore content (58.9 %) observed at 2 h and the highest mesopore content (13.5 %) at 4 h. However, the 5 h treatment reduced both macro- and mesopores while increasing micropore content, indicating that excessive treatment may lead to structural collapse or re-deposition of extractives. Overall, moderate ultrasonic treatment (1–4 h) effectively disrupted pit membranes, removed cell wall deposits, and enhanced pore connectivity, thereby increasing macropore and mesopore fractions, porosity, and cumulative pore volume, ultimately improving wood permeability. In contrast, overlong treatment (5 h) may compromise these benefits due to structural damage. Therefore, optimizing ultrasonic treatment duration based on application requirements is critical to maximizing pore structure improvement.

Ultrasonic pretreatment significantly enhanced the permeability of both *C. lanceolata* and *E. grandis × urophylla*, with radial values consistently higher than tangential due to anatomical features such as rays and vessels [39]. For *C. lanceolata*, radial permeability increased from 1.35 × 10^−9^ m^2^ (0 h) to 2.20 × 10^−9^ m^2^ (+62.96 %) at 300 min, and tangential permeability rose from 1.08 × 10^−9^ m^2^ to 1.72 × 10^−9^ m^2^ (+59.63 %). These improvements likely stem from pit membrane disruption and enhanced pore connectivity. A fluctuating “increase–decrease-increase” pattern was observed, suggesting dynamic structural changes during treatment. *E. grandis × urophylla* showed lower baseline permeability (0.72 × 10^−9^ m^2^ radial; 0.65 × 10^−9^ m^2^ tangential) but also responded positively. Peak radial permeability reached 1.02 × 10^−9^ m^2^ (+41.67 %) at 180 min, while tangential peaked at 0.87 × 10^−9^ m^2^ (+33.33 %) at 210 min. Permeability declined slightly at prolonged durations, possibly due to cell wall collapse or re-deposition of extractives [19,21]. In summary, moderate ultrasonic durations (e.g., 300 min for *C. lanceolata*, 180–210 min for *E. grandis × urophylla*) optimized permeability. Overexposure may compromise structural integrity, underscoring the need for species-specific treatment parameters.

### 3.4 Drying performance

**Fig. 9:**
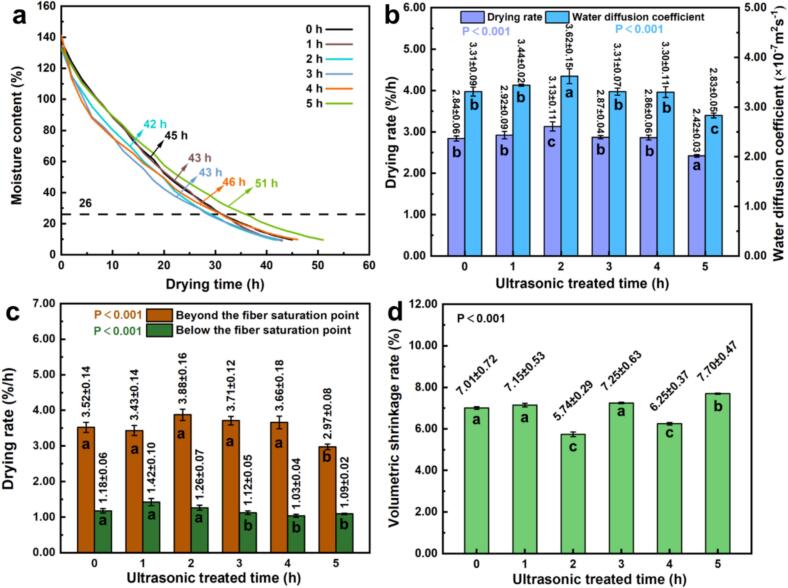
Effects of ultrasonic pretreatment duration on drying kinetics and dimensional stability of *C. lanceolata.*

**Fig. 10:**
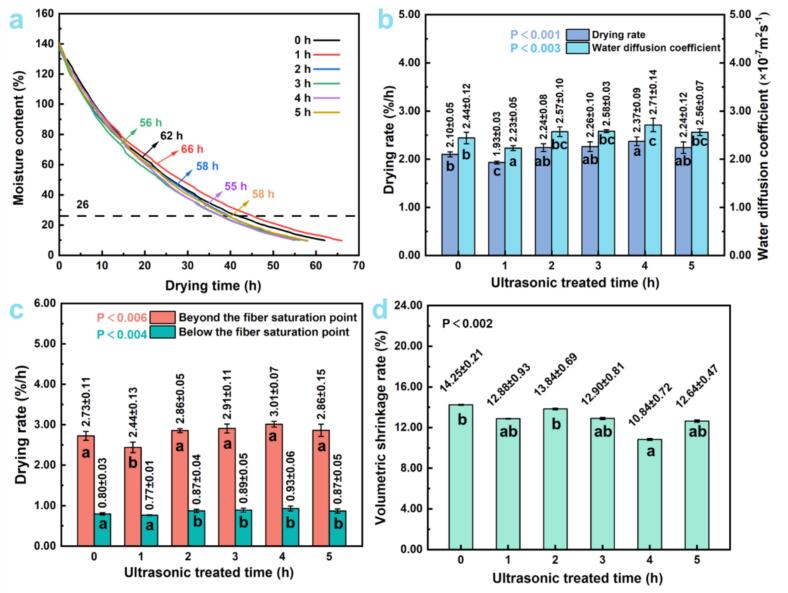
Effects of ultrasonic pretreatment duration on drying kinetics and dimensional stability of *E. grandis × urophylla.*

Fig. 9, Fig. 10 present the changes in moisture content (MC) over time during drying for *Cunninghamia lanceolata* and *Eucalyptus grandis × urophylla*, respectively. Ultrasonic pretreatment notably affected drying duration in both species, with a nonlinear response depending on treatment time. For *C. lanceolata* (Fig. 9a), untreated samples took 45 h to dry from 140 % to 10 % MC. Drying duration was reduced to 43 h after 1–3 h of pretreatment and reached the minimum (42 h) at 2 h. However, prolonged treatment (4–5 h) extended drying time to 46 h and 51 h, respectively, likely due to structural damage or pore collapse [23,40]. *E. grandis × urophylla* (Fig. 10a) exhibited a more complex pattern: the drying time increased to 66 h after 1 h of pretreatment but decreased to 55 h at 4 h, representing the greatest improvement (-7 h). A further increase to 58 h was observed at 5 h.

Average drying rates followed a “rise-fall” trend in both species. For *C. lanceolata* (Fig. 9b), the untreated rate was 2.84 %/h, peaking at 3.13 %/h after 2 h (+0.29 %/h), but dropping to 2.42 %/h at 5 h. Corresponding diffusion coefficients rose from 3.31 × 10^−7^ m^2^/s (control) to 3.62 × 10^−7^ m^2^/s (+9.37 %) at 2 h and declined to 2.83 × 10^−7^ m^2^/s at 5 h. For *E. grandis × urophylla* (Fig. 10b), the untreated rate was 2.10 %/h; it dropped to 1.93 %/h at 1 h, then increased to a maximum of 2.37 %/h at 4 h. Diffusion coefficients followed a similar trend, peaking at 2.71 × 10^−7^ m^2^/s (+11.07 %) at 4 h, suggesting that ultrasonic treatment improves the moisture mobility through the cell wall and lumen network [23,41].

Moisture migration was further analyzed above and below the fiber saturation point (FSP, 26 %). In *C. lanceolata* (Fig. 9c), the highest drying rate above FSP occurred at 3 h (3.71 %/h), while the fastest below FSP was at 1 h (1.42 %/h). In *E. grandis × urophylla* (Fig. 10c), the 4 h treatment achieved the highest rates in both phases (3.01 %/h above FSP, 0.93 %/h below), outperforming all other treatments. Volume shrinkage (Fig. 9d, 10d) decreased with moderate ultrasonic exposure. *C. lanceolata*’s shrinkage dropped from 7.01 % (untreated) to a minimum of 5.74 % at 2 h but increased to 7.70 % at 5 h. In *E. grandis × urophylla*, shrinkage was highest in untreated samples (14.25 %) and lowest at 4 h (10.84 %), a reduction of 3.41 %. Reduced shrinkage suggests a more stable drying process with less structural stress. These results indicate that moderate ultrasonic pretreatment improves water diffusivity, drying rate, and dimensional stability. Optimal durations were 2 h for *C. lanceolata* and 4 h for *E. grandis × urophylla*. Over- or under-treatment may impair drying performance, highlighting the importance of species-specific optimization in industrial applications.

## 4 Conclusion

This study systematically investigated the effects of different ultrasonic treatment durations (1–5 h) on the physicochemical properties, microstructure, permeability, and drying behavior of *Cunninghamia lanceolata* and *Eucalyptus grandis × urophylla*. The results showed that ultrasonic treatment significantly increased weight loss and microfibril angle, reduced crystallinity and the content of free hydroxyl groups, and improved hygroscopic and dimensional stability. Cell wall thickness and Shore hardness across all sections exhibited a pattern of initial decrease, stabilization, and subsequent decline with prolonged treatment. Microscopic observation and MIP analysis revealed that ultrasound progressively disrupted pit membranes, removed vessel deposits, and expanded pore channels, thereby increasing the proportion of macro- and mesopores, total pore volume, and porosity, which significantly enhanced both radial and tangential gas permeability. The maximum increases reached 63 % and 60 %, respectively. However, excessive treatment (5 h) may lead to structural collapse or mechanical deterioration, reducing pore quality. Drying experiments indicated that ultrasonic treatment promoted water migration, especially of free water, shortened drying time, increased drying rate and diffusion coefficient, and reduced volumetric shrinkage. The optimal drying performance was observed at 2 h for *C. lanceolata* and 4 h for *E. grandis × urophylla*, with drying rates increased by approximately 10 % and 13 %, respectively. In contrast, bound water migration was less responsive to ultrasonic stimulation, suggesting a threshold beyond which additional treatment may not improve effectiveness. Overall, appropriate ultrasonic treatment can synergistically optimize wood microstructure and moisture pathways, significantly enhancing permeability and drying efficiency, thus offering theoretical and technical support for industrial drying and permeability modification of fast-growing plantation wood.

## CRediT authorship contribution statement

**Jing Qian:** Writing – review & editing, Writing – original draft, Visualization, Validation, Supervision, Software, Resources, Project administration, Methodology, Funding acquisition, Formal analysis, Conceptualization. **Taoyu Han:** Software, Investigation, Data curation. **Taorong Cheng:** Software, Investigation, Data curation. **Pengfei Xia:** Investigation. **Zekai Sun:** Investigation. **Chunping Dai:** Writing – review & editing. **Jiejie Sun:** Writing – review & editing.

## Declaration of competing interest

The authors declare that they have no known competing financial interests or personal relationships that could have appeared to influence the work reported in this paper.

## References

[1] Elustondo D., Matan N., Langrish T., Pang S. (2023). Advances in wood drying research and development. Drying Technol..

[2] Onu Olughu O., Tabil L.G., Dumonceaux T. (2021). Ultrasonic delignification and microstructural characterization of switchgrass. Energies.

[3] Petty J.A., Preston R.D. (1970). Permeability and structure of the wood of Sitka spruce. Proc. R. Soc. Lond. B.

[4] Thybring E.E., Fredriksson M., Zelinka S.L., Glass S.V. (2022). Water in wood: a review of current understanding and knowledge gaps. Forests.

[5] Meyer R.W., Barton G.M. (1971). A relationship between collapse and extractives in Western Red Cedar. Forest Prod. J..

[6] Cocusse M., Rosales M., Maillet B., Sidi-Boulenouar R., Julien E., Caré S., Coussot P. (2022). Two-step diffusion in cellular hygroscopic (vascular plant-like) materials. Sci. Adv..

[7] Simpson W.T. (1973). Predicting equilibrium moisture content of wood by mathematical models. Wood Fiber Sci..

[8] Jakes J.E., Hunt C.G., Zelinka S.L., Ciesielski P.N., Plaza N.Z. (2019). Effects of moisture on diffusion in unmodified wood cell walls: a phenomenological polymer science approach. For.

[9] Lehringer C., Richter K., Schwarze F.W., Militz H. (2009). A review on promising approaches for liquid permeability improvement in softwoods. Wood Fiber Sci..

[10] Nicholas D.D. (1977). Chemical methods of improving the permeability of wood. Am. Chem. Soc. Symp. Ser..

[11] Y. Qin, Improvement of Eucalyptus urophylla wood permeability via urea treatment, Bioresour. 18(3) (2023).4790-4804.

[12] Hermawan A., Priadi T., Murano T., Sakagami H., Fujimoto N. (2024). High-temperature drying: a review of fundamental research and development in wood drying. Dry. Technol..

[13] He X., Xiong X., Xie J., Li Y., Wei Y., Quan P., Mou Q., Li X. (2017). Effect of microwave pretreatment on permeability and drying properties of wood. Bioresource.

[14] Deng X., Peng W., Wu X., Xiao F., Ye C., Li K. (2024). Effect of tracheid on water absorption behavior of *Cunninghamia lanceolata* under freeze-thaw conditions. Eur. J. Wood Wood Prod..

[15] Statnikov E.S., Korolkov O.V., Vityazev V.N. (2006). Physics and mechanism of ultrasonic impact. Ultrason.

[16] Yao Y. (2016). Enhancement of mass transfer by ultrasound: Application to adsorbent regeneration and food drying/dehydration. Ultrason. Sonochem..

[17] Ong V.Z., Wu T.Y. (2020). An application of ultrasonication in lignocellulosic biomass valorisation into bio-energy and bio-based products. Renew. Sust. Energ. Rev..

[18] Kumar K., Srivastav S., Sharanagat V.S. (2021). Ultrasound assisted extraction (UAE) of bioactive compounds from fruit and vegetable processing by-products: a review. Ultrason. Sonochem..

[19] Han T., Lu M., Cui S., Liu S., Avramidis S., Qian J. (2023). How does ultrasound contribute to the migration of extractives inside Ailanthus altissima wood?. Ultrason. Sonochem..

[20] Qian J., Gao J., Zhao F., He L., Zhang T., He Z., Yi S. (2021). How does ultrasound influence the thermal stability of wood?. Ind. Crop Prod..

[21] Qian J., Zhao F., Gao J., Qu L., He Z., Yi S. (2021). Characterization of the structural and dynamic changes of cell wall obtained by ultrasound-water and ultrasound-alkali treatments. Ultrason. Sonochem..

[22] He Z., Zhang Y., Wang Z., Zhao Z., Yi S. (2016). Reducing wood drying time by application of ultrasound pre-treatment. Dry Technol..

[23] H. Liu, Y. Zhang, L. Yang, Z.H. Wu, Effects of ultrasound pretreatment on microstructure and drying characteristics of Eucalyptus urophylla× E. grandis, Bioresource 13(3) (2018) 5953-5964. Doi: 10.15376/biores.13.3.5953-5964.

[24] Segal L., Creely J.J., Martin A.E., Conrad C.M. (1959). An empirical method for estimating the degree of crystallinity of native cellulose using the X-ray diffractometer. Text. Res. J..

[25] T.J. Mason, J.P. Lorimer, Applied sonochemistry: the uses of power ultrasound in chemistry and processing. In Applied Sonochemistry: The Uses of Power Ultrasound in Chemistry and Process. Wiley-Blackwell, 2002, pp. 1-293.

[26] Gogate P.R., Pandit A.B. (2004). A review of imperative technologies for wastewater treatment I: oxidation technologies at ambient conditions. Adv. Environ. Rres..

[27] Flores E.M., Cravotto G., Bizzi C.A., Santos D., Iop G.D. (2021). Ultrasound-assisted biomass valorization to industrial interesting products: state-of-the-art, perspectives and challenges. Ultrason. Sonochem..

[28] Baruah J., Nath B.K., Sharma R., Kumar S., Deka R.C., Baruah D.C., Kalita E. (2018). Recent trends in the pretreatment of lignocellulosic biomass for value-added products. Front. Energy Res..

[29] Wang P., Liu C., Chang J., Yin Q., Huang W., Liu Y., Dang X., Gao T., Xu F. (2019). Effect of physicochemical pretreatments plus enzymatic hydrolysis on the composition and morphologic structure of corn straw. Renew. Energ..

[30] Sun Y., Liu L., Deng H., Li J., He B., Sun R., Ouyang P. (2008). Structural changes of bamboo cellulose in formic acid. Bioresour.

[31] Peng H., Jiang J., Zhan T., Lu J. (2016). Influence of density and equilibrium moisture content on the hardness anisotropy of wood. Forest Prod. J..

[32] Nagula K.N., Pandit A.B. (2016). Process intensification of delignification and enzymatic hydrolysis of delignified cellulosic biomass using various process intensification techniques including cavitation. Bioresour. Technol..

[33] Qian J., Li Y., Gao J., He Z., Yi S. (2020). The effect of ultrasonic intensity on physicochemical properties of Chinese fir. Ultrason. Sonochem..

[34] Wang C., Jiang Z., Fei B., Yu Y., Zhang S. (2012). Effects of chemical components on longitudinal MOE and hardness of wood cell wall. J. Beijing for. Univ..

[35] Salmén L. (2004). Micromechanical understanding of the cell-wall structure. C. R. Biol..

[36] Chen T., Liu R., Zhang Y., Shen Y., Yao L., Wang X. (2025). Ultrasonic-activated persulfate treatment for enhancing pore volume and permeability of poplar wood. Ind. Crops Prod..

[37] Tanaka T., Avramidis S., Shida S. (2010). A preliminary study on ultrasonic treatment effect on transverse wood permeability. Maderas, Ciencia y Tecnología.

[38] Chen Y., Choong E.T. (1994). Determining the effect of extractives on moisture movement using a “continuous” measuring system. Wood Fiber Sci..

[39] Siau J.F. (1984).

[40] Plötze M., Niemz P. (2011). Porosity and pore size distribution of different wood types as determined by mercury intrusion porosimetry. Eur. J. Wood Wood Prod..

[41] Thybring E.E., Fredriksson M. (2021). Wood modification as a tool to understand moisture in wood. Forests.

