# Supplementary material for: Effects of ultrasonic pretreatment on physicochemical properties and drying behavior of Cunninghamia lanceolata and Eucalyptus grandis × urophylla
Source: Ultrason Sonochem. 2025 Sep 3;121:107549. doi: 10.1016/j.ultsonch.2025.107549 (PMC12504980; doi:10.1016/j.ultsonch.2025.107549)
Supplement: Supplementary Data 1 [file mmc1.docx]

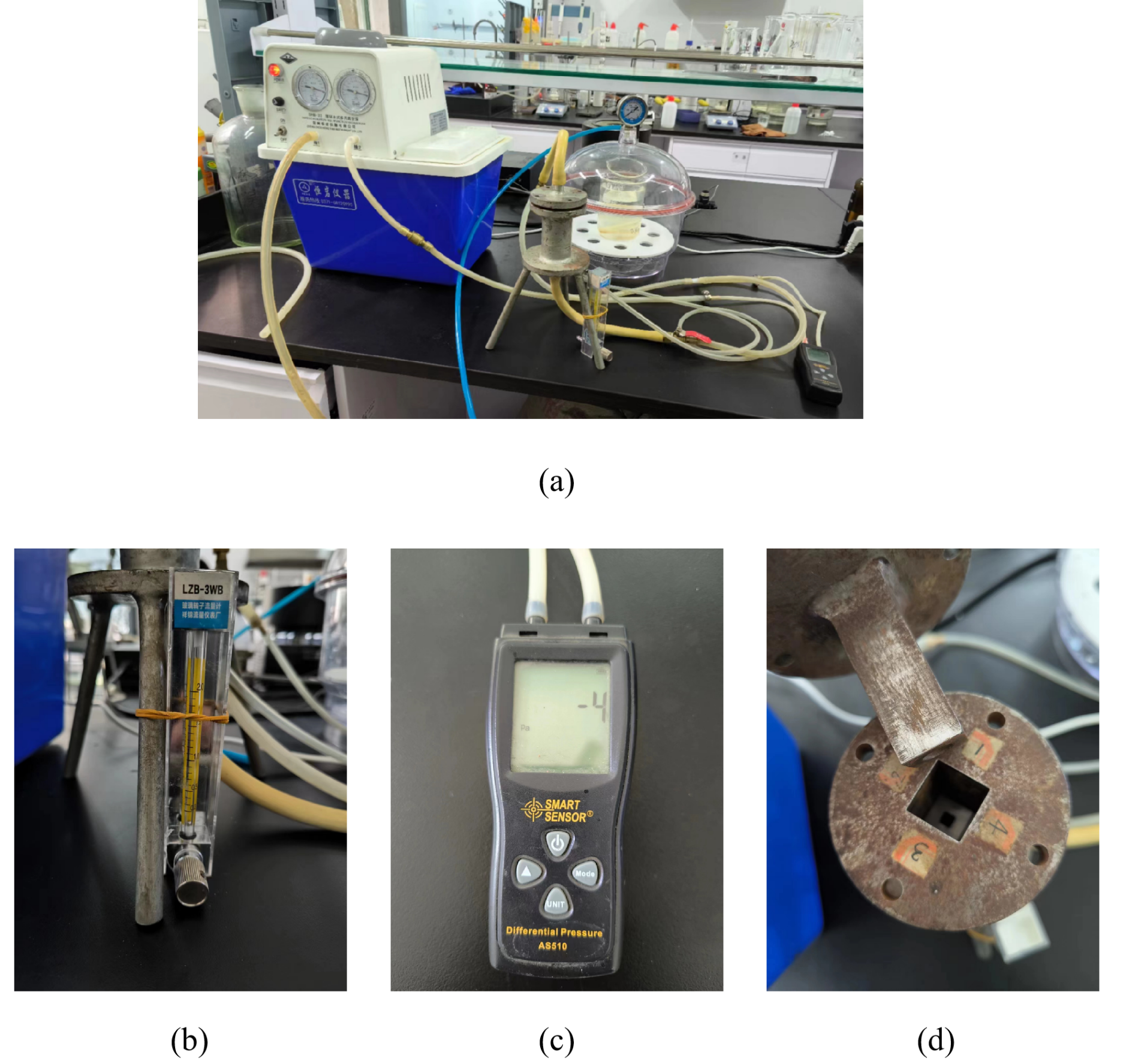


Fig. 1 Diagram of the permeability measuring apparatus: (a) permeability measuring device; (b) flow meters; (c) differential pressure gauge; (d) specimen clamping assembly
